# A comparative analysis of metabarcoding and morphology‐based identification of benthic communities across different regional seas

**DOI:** 10.1002/ece3.4283

**Published:** 2018-08-13

**Authors:** Abigail E. Cahill, John K. Pearman, Angel Borja, Laura Carugati, Susana Carvalho, Roberto Danovaro, Sarah Dashfield, Romain David, Jean‐Pierre Féral, Sergej Olenin, Andrius Šiaulys, Paul J. Somerfield, Antoaneta Trayanova, Maria C. Uyarra, Anne Chenuil

**Affiliations:** ^1^ Institut Méditerranéen de Biodiversité et d'Ecologie marine et continentale (IMBE) Aix Marseille Univ Avignon Université, CNRS IRD IMBE Marseille France; ^2^ Biology Department Albion College Albion Michigan USA; ^3^ King Abdullah University of Science and Technology (KAUST) Red Sea Research Center Thuwal Saudi Arabia; ^4^ AZTI Marine Research Division Herrera Kaia Pasaia Spain; ^5^ Dipartimento di Scienze della Vita e dell'Ambiente Università Politecnica delle Marche Ancona Italy; ^6^ Stazione Zoologica “A. Dohrn”, Villa Comunale Napoli Italy; ^7^ Plymouth Marine Laboratory Plymouth UK; ^8^ Marine Research Institute Klaipėda University Klaipėda Lithuania; ^9^ Nikola Vaptsarov Naval Academy Varna Bulgaria; ^10^ Institute of Oceanology (IO‐BAS) Bulgarian Academy of Sciences Varna Bulgaria

**Keywords:** Artificial Substrate Unit (ASU), COI, innovative monitoring, marine invertebrates, metabarcoding

## Abstract

In a world of declining biodiversity, monitoring is becoming crucial. Molecular methods, such as metabarcoding, have the potential to rapidly expand our knowledge of biodiversity, supporting assessment, management, and conservation. In the marine environment, where hard substrata are more difficult to access than soft bottoms for quantitative ecological studies, Artificial Substrate Units (ASUs) allow for standardized sampling. We deployed ASUs within five regional seas (Baltic Sea, Northeast Atlantic Ocean, Mediterranean Sea, Black Sea, and Red Sea) for 12–26 months to measure the diversity and community composition of macroinvertebrates. We identified invertebrates using a traditional approach based on morphological characters, and by metabarcoding of the mitochondrial cytochrome oxidase I (COI) gene. We compared community composition and diversity metrics obtained using the two methods. Diversity was significantly correlated between data types. Metabarcoding of ASUs allowed for robust comparisons of community composition and diversity, but not all groups were successfully sequenced. All locations were significantly different in taxonomic composition as measured with both kinds of data. We recovered previously known regional biogeographical patterns in both datasets (e.g., low species diversity in the Black and Baltic Seas, affinity between the Bay of Biscay and the Mediterranean). We conclude that the two approaches provide complementary information and that metabarcoding shows great promise for marine monitoring. However, until its pitfalls are addressed, the use of metabarcoding in monitoring of rocky benthic assemblages should be used in addition to classical approaches rather than instead of them.

## 1 INTRODUCTION

To effectively conserve biodiversity at all levels of biological organization, the first crucial step is monitoring and assessment (Patrício et al., 2016). However, monitoring in some habitats remains difficult (Carugati, Corinaldesi, Dell'Anno, & Danovaro, 2015). The hard‐bottom subtidal zone of the marine environment can be monitored using technologically advanced, often costly, methods (e.g., *in situ* chambers and equipment) or time‐consuming scientific diving. Thus, our knowledge about the effects of human pressures on these communities is still limited. Increasing this understanding is a priority, and requires both implementing innovative measures to monitor marine biodiversity and developing standardized protocols (Danovaro et al., 2016).

In addition, identifying the species present in subtidal habitats is not always easy. Monitoring hard‐bottom organisms typically requires the morphological identification of species. This method requires specialized expertise and is too time‐consuming and costly for routine monitoring, especially at large scales (Carugati et al., 2015; Ferraro, Cole, DeBen, & Swartz, 1989; McManus & Katz, 2009). The use of traditional taxonomy is also complicated by the presence of cryptic species, which are genetically distinct but morphologically indistinguishable (Knowlton, 1993, 2000), or by cryptic developmental stages (Pfenninger & Schwenk, 2007).

Alternatively, molecular metabarcoding has been proposed as a promising method to rapidly measure the community composition based on the genetic identification of species in an area (Bourlat et al., 2013; Cristecu, 2014; Taberlet, Coissac, Pompanon, Brochmann, & Willerslev, 2012). Recent studies have quantified biodiversity using metabarcoding techniques in many habitats (e.g., Andersen et al., 2012; Yu et al., 2012). Molecular data may also be able to identify members of the community that are present in the guts of larger organisms, which otherwise would be impossible to identify based on morphology. In recent years, molecular metabarcoding has been increasingly recognized for its potential contribution to the study of marine biodiversity (e.g., Brannock, Ortmann, Moss, & Halanych, 2016; Bucklin, Lindeque, Rodriguez‐Ezpeleta, Albaina, & Lehtiniemi, 2016; Kelly et al., 2017; Lejzerowicz et al., 2015; Leray & Knowlton, 2015; Pearman, Anlauf, Irigoien, & Carvahlo, 2016; de Vargas et al., 2015). Molecular techniques and the use of a single barcoding gene allow for rapid identification of specimens in marine communities (Danovaro et al., 2016). Although metabarcoding is a highly promising technique, it has its drawbacks as well, including sensitivity of the results to marker choice and the fact that reference databases are incomplete (Carugati et al., 2015; Danovaro et al., 2016; Deagle, Jarman, Coissac, Pompanon, & Taberlet, 2014; Deiner et al., 2017).

Standardized sampling methods and analytical protocols and techniques for marine habitats are highly desirable for reliable descriptions of biodiversity and community composition (Hering et al., 2018). Hard‐bottom marine substrata cannot be sampled using the same methods that have been developed in other habitats (e.g., grabs, Danovaro et al., 2016). To standardize sampling in these areas, Artificial Substrate Units (ASUs) such as nylon pan scourers can provide a standardized volume and have been used to quantitatively sample early life stages of target taxa, or to experimentally manipulate and sample whole communities (Gobin & Warwick, 2006; Hale, Calosi, McNeill, Mieszkowska, & Widdicombe, 2011; Kendall et al., 1996; Menge, Berlow, Blanchette, Navarrete, & Yamada, 1994; Menge, Chan, Nielsen, Di Lorenzo, & Lubchenco, 2009; Menge et al., 2002; Underwood & Chapman, 2006). These ASUs mimic algal holdfasts or seagrasses (Kendall et al., 1996; Menge et al., 1994; Paine, 1974) and the small mesh size allows for the sampling of small‐bodied taxa. ASUs may therefore target a different set of taxa than would be sampled when using hard settlement plates or Autonomous Reef Monitoring Structures (ARMS) (e.g., Leray & Knowlton, 2015; Pearman et al., 2016; Pearman et al., 2018). Other studies, including some conducted in the marine environment, have used morphology and metabarcoding to analyze communities (e.g., Cowart et al., 2015; Kelly et al., 2017; Lejzerowicz et al., 2015), but few studies exist that compare these methods in hard‐bottom environments.

**Figure 1:**
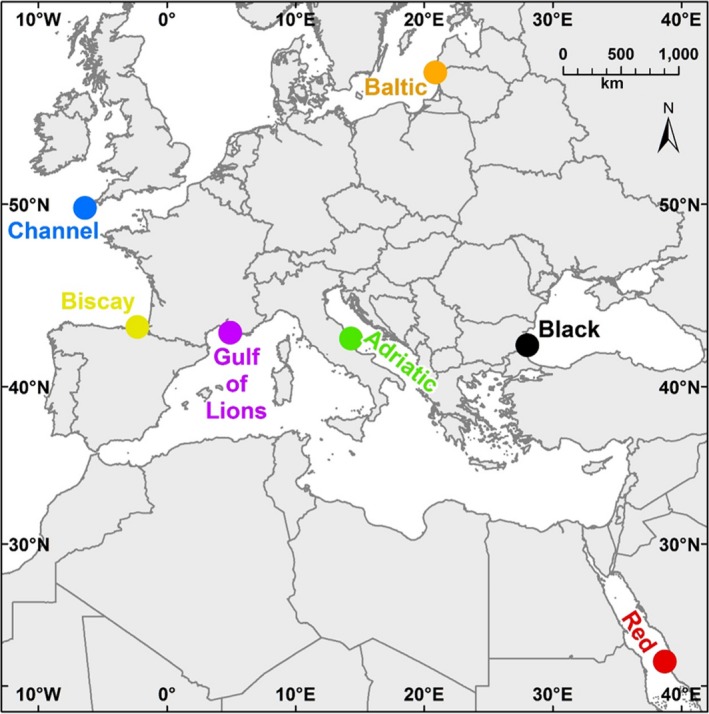
Map of sampling locations. The seven locations within five regional seas sampled in this study. Locations were sampled at multiple sites, with multiple artificial sampling units per site. Complete sampling information is listed in Table 1 [Colour figure can be viewed at http://wileyonlinelibrary.com]

In this study, we use ASUs and both metabarcoding and traditional morphological analysis to explore benthic communities. Our goals were both to compare metabarcoding and morphological analysis in assessing benthic diversity patterns and to evaluate the suitability of using our sampling and analysis protocols in several regional seas. Sampling was undertaken in seven geographically widespread locations (Table 1, Figure 1), and we used both morphological and molecular methods to identify the macroinvertebrates found in these locations. We chose the mitochondrial gene COI as our barcoding gene, as it is one of the preferred loci for “universal” barcoding (Lorenz, Jackson, Beck, & Hanner, 2005), has a large reference database, is highly variable between species, and has been already used in previous studies to assess benthic metazoan biodiversity (e.g., Leray & Knowlton, 2015). Both methods (morphological and molecular) were used to measure taxonomic richness and diversity and community composition with the hope of making recommendations for future monitoring programs. In addition, our sampling design allowed us to evaluate the effectiveness of the two methods in distinguishing biogeographic patterns among regions and whether or not these methods are viable in a wide range of seas.

**Table 1:** Sampling sites. Details of sampling sites, including the location, site name, depth of deployment, dates of deployment and recovery, and the number of Artificial Substrate Units (ASUs) recovered. All sites started with 3 ASUs

Location	Site	Latitude	Longitude	Depth (m)	Date deployed	Date recovered	*N* recovered
Baltic Sea	Karkle	55°47.352 N	21°2.518 E	8	June 2013	August 2015	1
Baltic Sea	Palanga	55°55.57 N	21°1.598 E	8	June 2013	August 2015	2
English Channel	Gugh Reef	49°53.180 N	06°19.345 W	19	May 2013	April 2014	2
Bay of Biscay	Lekeitio	43°22.311 N	2°30.258 W	12	June 2013	July 2014	1
Bay of Biscay	Pasaia	43°20.231 N	1°55.638 W	11	May 2013	May 2014	2
Bay of Biscay	Zumaia	43°18.748 N	2°13.641 W	11	May 2013	June 2014	3
Gulf of Lions	Cassidaigne	43°8.740 N	5°32.740 E	17	July 2013	December 2014	3
Gulf of Lions	Elvine	43°19.780 N	5°14.210 E	17	June 2013	December 2014	3
Gulf of Lions	Rioux Sud	43°10.370 N	5°23.420 E	17	June 2013	December 2014	3
Adriatic Sea	Due Sorelle	43°32.953 N	13°37.699 E	9	June 2014	July 2015	3
Adriatic Sea	Grotta Azzurra	43°37.313 N	13°31.691 E	7	June 2014	July 2015	2
Adriatic Sea	La Scalaccia	43°36.291 N	13°33.102 E	9	June 2014	July 2015	2
Black Sea	Aladja Bank	43°16.800 N	28°03.396 E	7	August 2013	September 2014	1
Black Sea	Cherni Nos	42°55.650 N	27°54.637 E	7	August 2013	September 2014	2
Black Sea	Kamchia	43°01.114 N	27°54.129 E	8	August 2013	September 2014	1
Red Sea	Janib Sa'ara Reef	21°27.253 N	39°06.661 E	10	April 2013	June 2014	1
Red Sea	Qaham Reef	21°04.921 N	39°12.063 E	10	April 2013	June 2014	3

## 2 METHODS

### 2.1 Artificial substrate units: deployment and recovery

ASUs were composed of four nylon pan scourers fastened together, attached to a stainless‐steel rod using a cable tie, and affixed to the substratum. We selected six sampling locations in five regional seas (Table 1, Figure 1). Within each of the six sampling locations, we chose three sites, with three ASUs deployed per site, for a total of nine per location. Samples were also available from a single site in the English Channel, our seventh location. Sites ranged from 7 to 19 m depth; most sites were between 7 and 12 m. ASUs were deployed between May 2013 and June 2014, and nearly all stayed in the field 12–14 months. Differences in deployment dates and lengths of deployment time are explained by weather and resource limitations that hindered boat and diving activity. Most notably, due to exceptional bad weather in the Baltic Sea, recovery of the samples in this location was not possible until after 26 months. The ASUs needed to be reinstalled in the Adriatic Sea following loss due to rough sea conditions. All ASUs were collected between May 2014 and August 2015. Divers recovered the ASUs, placing them in containers at the collection site underwater to prevent loss of material and returned immediately to the laboratory, where the samples were stored in ethanol (except for those from the English Channel, which were stored in formalin). Not all replicates were recovered at all sites. Table 1 contains the complete sampling and location information.

### 2.2 Morphological data collection

In the laboratory, we separated the four pan scourers that made up each ASU and removed the mobile animals. We shook each scourer vigorously in deionized water to remove loose material, and then cut it open to pick out material that remained stuck in the mesh. We sieved the material from each scrubber on a 40 μm mesh and visually sorted it to collect animals larger than approximately 1 mm, which were then preserved in ethanol. Following the sorting procedure, specimens were identified to a standard taxonomic level (usually class) based on morphological characters and we counted the number of individuals belonging to each taxonomic group. The full list of groups identified with morphological sorting is available in Supporting Information Appendix S1. A single person did the sorting to minimize observer bias, but this limited our ability to identify taxa more precisely over the large geographic scale of the study. Within each taxonomic group, we focused on the lowest level of classification that could easily and rapidly be identified. Limiting the taxonomic resolution at this step limits the precision of biological conclusions that we can draw from our data, but allowed us to compare data collected with morphological and molecular methods given a roughly equal time investment. After identification, specimens were pooled into phylum‐level groups, and the biomass for each group was measured. Five groups were used for each sample: annelids, arthropods, echinoderms, molluscs, and “other” (animals that did not fit into one of the four preceeding groups).

### 2.3 Metabarcoding protocol

All samples were then analyzed using a metabarcoding approach (excepting those from the English Channel, which had been stored in formalin). After calculating biomass, the phylum‐level groups were ground using a mortar and pestle. Phylum‐specific extractions were used to reduce overrepresentation of large‐bodied (Elbrecht, Peinert, & Leese, 2017) or extremely common organisms in the sequencing (e.g., amphipods in the Bay of Biscay). We extracted DNA from up to 0.4 g of mixed tissue using Machery‐Nagel NucleoSpin^®^ 96 Tissue Kits. Separate extractions were performed for each phylum of each sample, for a total of 151 individual extractions. The amount of DNA in each extraction was quantified with fluorometry using a Qubit 2.0 (Invitrogen).

We pooled the DNA from all phyla for a single sample in equimolar concentrations (i.e., most samples contained DNA from five different extractions) and quantified the DNA in the pools using a Qubit 2.0. We chose to use the mitochondrial gene COI as our barcoding gene, due to its large reference database and other reasons described above. We used PCR to amplify the mitochondrial (mt) COI barcodes from the pools, using approximately 5 ng DNA, 10 μl Phusion^®^ High‐Fidelity master mix (New England BioLabs), and 0.4 μl each of the forward and reverse primers for each 20 μl reaction. We used primers from Leray et al. (2013), which were developed for metabarcoding of metazoans (Leray & Knowlton, 2015; Leray et al., 2013). We conducted three replicate PCRs on each sample pool using the following PCR program: 3 min at 98°C, 27 cycles (10 s 98°C, 30 s 46°C, 45 s 72°C), 5 min at 72°C. We verified amplification for each replicate visually on a 1.5% agarose gel, pooled the replicates together, and then sent the pooled PCR product to the ICM‐Brain and Spine Institute (Paris, France) for final library preparation prior to sequencing. This preparation included a second PCR for the addition of adapters used in Illumina sequencing; libraries were prepared using a TruSeq HT kit. Negative controls were run during the PCR, but due to the lack of DNA in these samples, they were not added to the sequencing run according to sequencing center protocols. Samples were sequenced using 250 bp paired‐end sequencing on an Illumina MiSeq. The raw sequences were deposited in the NCBI Short Read Archive (SRA) under the accession number SRP093498.

### 2.4 Bioinformatic analysis

Raw reads from the sequencing run were automatically demultiplexed. The paired ends were joined with a minimum of 50 bp and a maximum difference of 10% in QIIME (Caporaso et al., 2010) and quality‐checked with split libraries using a Phred score of 24. Further quality filtering and the removal of primers from the reads was undertaken in mothur (Schloss et al., 2009) using trim.seqs (pdiffs = 0, maxhomop = 8, maxambig = 0). Using the trie function in pick_otus.py (QIIME), unique sequences were produced. The reference sequences produced in this step were aligned and preclustering was undertaken in mothur (diffs = 3). Singletons were removed (split.abund with a cutoff of 1 in mothur) and chimeras removed using u‐search (Edgar, 2010). Lastly, molecular operational taxonomic units (mOTUs) based on similarity (97%) were produced using usearch (in QIIME's pick_otus.py). Reference sequences for the mOTUs were assigned a taxonomy against the BOLD database (Ratnasingham & Hebert, 2007) using the Ribosomal Database Project method (rdp; Wang, Garrity, Tiedje, & Cole, 2007; confidence 0.5) within the assign_taxonomy script in QIIME. The assigned mOTUs were checked by eye for obvious contamination. Two mOTUs belonging to the Antarctic urchin genus *Abatus* were identified. DNA from this genus was being handled in the laboratory at the same time as the ASUs samples, so these mOTUs were classified as contamination and removed from the dataset. The number of reads per sample was rarefied multiple times (*n* = 100) at a depth of 8,200 reads within the QIIME framework and an mOTU table produced for diversity analyses.

### 2.5 Statistical analysis

We compared community composition based on morphological identification among the seven different locations. We conducted a permutational multivariate analysis of variance (PERMANOVA) with the data, with sites nested within locations, as well as a non‐metric multidimensional scaling (NMDS) analysis based on Bray–Curtis distances. Data were fourth‐root transformed prior to these analyses to reduce the influence of very common taxa (as in Clarke, 1993). We compared community richness (Margalef's Index, d’) and diversity (Simpson's Index, 1‐lambda’) among locations using nested PERMANOVAs, again with sites nested within locations. NMDS analyses were conducted using the vegan package, version 2.4‐0 (Oksanen et al., 2016), with R version 3.3.1 (R Core Team 2016), and PERMANOVAs were conducted with the PERMANOVA+ package in PRIMER (Anderson, Gorley, & Clarke, 2008; Clarke & Gorely, 2015; Clarke, Gorely, Somerfield, & Warwick, 2014).

The same analyses were conducted on the metabarcoding data from six locations (excluding the English Channel samples). First, we conducted all analyses based on the mOTU table. Second, for a more direct comparison to the results obtained from morphological identifications, we collapsed the mOTU list to match the morphological data (usually to the class level) by taking the sum of all reads in each higher taxonomic group, removed unclassified OTUs as they did not match any morphological identification, and conducted all analyses again. This analysis also allowed us to compare the two datasets while accepting similar amounts of error: Porter and Hajibabei (2018) found that taxa were assigned to the correct order, class, or phylum 99% of the time (i.e., 99% accuracy) when using COI barcodes of ~400 bp length. The collapsed analyses are therefore direct comparisons to the morphological dataset both in terms of the categories used in the analysis and in the amount of error in the dataset (both are highly accurate). The full mOTU table, along with the higher taxonomic designations used for the collapsed analyses, is available in Supporting Information Appendix S2. We correlated the diversity measures calculated with the morphological and molecular data (all mOTUs considered).

We also collapsed the mOTUs to the phylum level by taking the sum of all reads in each phylum and correlated the number of reads recovered with the biomass for each phylum. Lastly, we tested the dissimilarity in composition between pairs of regions by computing dissimilarity matrices using Bray–Curtis distances. We calculated the matrices from the molecular and morphological data using the Relate function (Mantel tests) in PRIMER (Clarke & Gorely, 2015; Clarke et al., 2014). Two tests were performed, one comparing the morphological data to the full set of mOTUs and one to the molecular data that had been collapsed to match the morphological data.

## 3 RESULTS

### 3.1 Morphological identification

**Figure 2:**
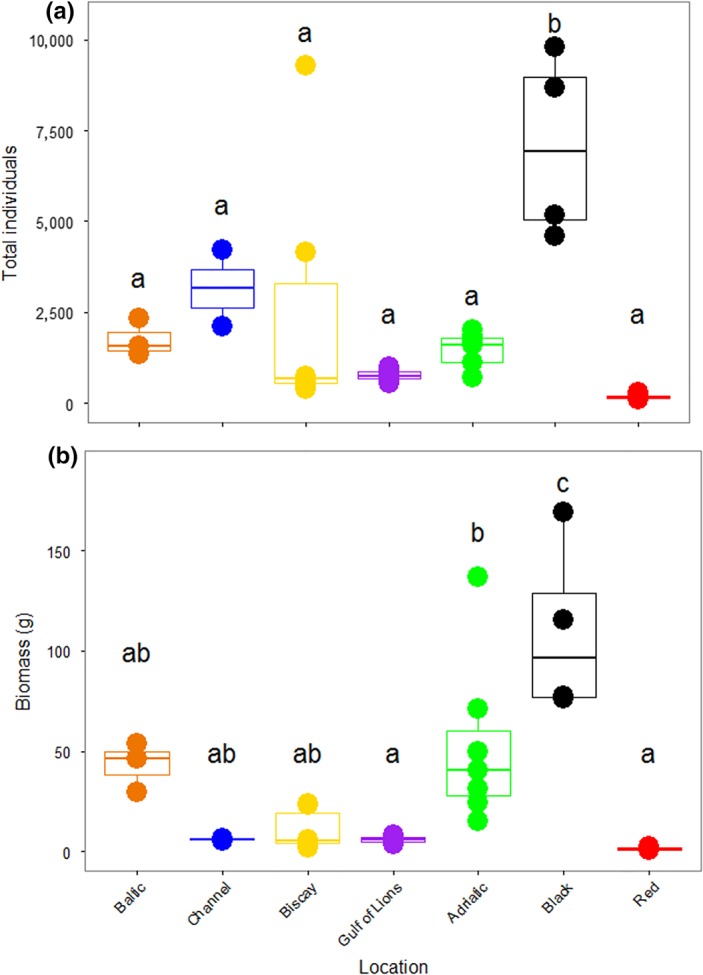
Total individuals and biomass removed from Artificial Substrate Units (ASUs). The total number of individuals (a) and biomass (b, in grams) removed from the ASUs in each of seven locations. Letters indicate significant differences among locations at the *p* < 0.05 level following Tukey's HSD tests and each point represents one ASU [Colour figure can be viewed at http://wileyonlinelibrary.com]

The number of specimens found in a single ASU ranged from 120 in the Red Sea to 9,787 in the Black Sea. There were significant differences in the number of organisms recovered among the different locations (*F*_6,28_ = 7.281, *p* < 0.001; Figure 2A). Tukey's HSD tests showed that the Black Sea ASUs contained significantly more individuals than all other locations. This was largely due to the preponderance of bivalves in the Black Sea (Supporting Information Figure S1A). The biomass of the organisms recovered from the ASUs was also different among locations (*F*_6,28_ = 11.45, *p* < 0.001; Figure 2B). Again, the bivalves in the Black Sea led to a greater biomass than in all other seas, as measured with Tukey's HSD. As biomass was measured including molluscan shells, these shells contributed to high total biomass. There was no correlation between the number of specimens found and the duration of deployment of the ASUs (*r* = −0.224, *p* = 0.2).

**Figure 3:**
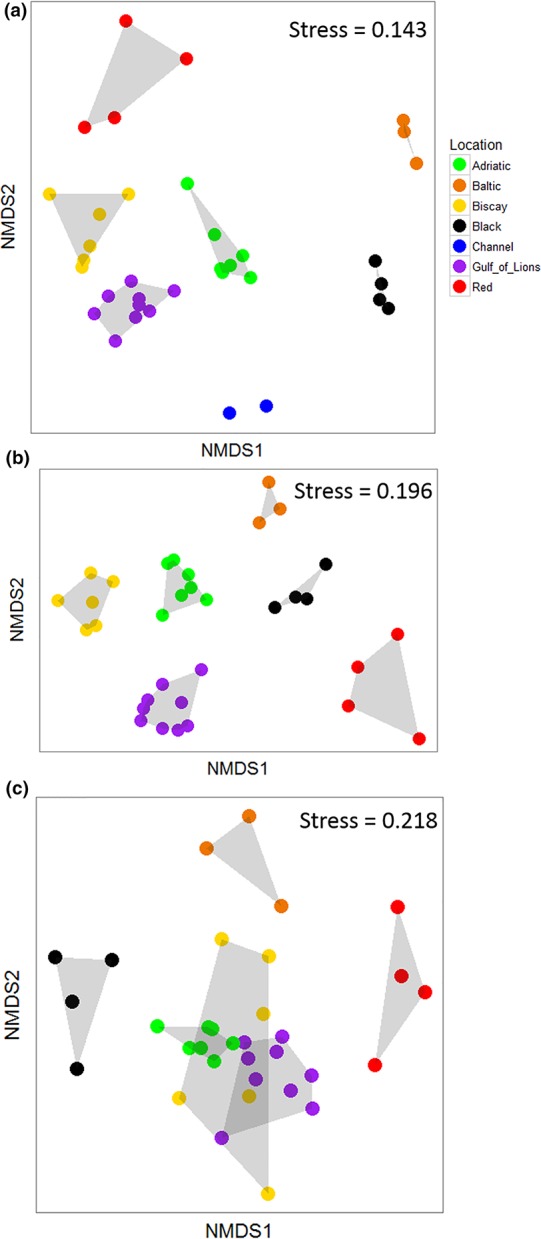
Community composition in different locations. Comparison of community composition among different locations using nonmetric multidimensional scaling analyses. Results are reported from (a) morphological identification, (b) molecular analyses (all molecular operational taxonomic units considered), (c) molecular analyses (data collapsed to match the morphological data) [Colour figure can be viewed at http://wileyonlinelibrary.com]

The community composition of the ASUs was significantly different both among locations (PERMANOVA, Table 2) and among sites nested within locations (PERMANOVA, Table 2). The NMDS analysis showed that locations with salinity >30 tended to cluster together, whereas the Black and Baltic Seas were separated on the plot (Figure 3A; stress = 0.143).

**Table 2:** Community composition. PERMANOVA comparing community composition within and among locations as measured with morphological identifications and with molecular data, both all molecular operational taxonomic units (mOTUs) considered (below left) and with mOTUs collapsed to match the morphological data (below right). Data were fourth‐root transformed prior to analysis. Significant effects at *p* < 0.05 are highlighted in bold

Morphological data				
Source of variation	*df*	MS	pseudo‐*F*	*p*
Location	**6**	**3,728.10**	**10.833**	**<0.001**
Sites (location)	**10**	**311.07**	**1.834**	**0.015**
Error	18	169.64		

Molecular data								
Source of variation	All mOTUs considered				mOTUs collapsed to match morphological data			
	*df*	MS	pseudo‐*F*	*p*	*df*	MS	pseudo‐*F*	*p*
Location	**5**	**12,291**	**4.109**	**<0.001**	**5**	**3,404.40**	**6.744**	**<0.001**
Sites (location)	**10**	**2,714.1**	**1.218**	**0.006**	10	455.46	0.980	0.530
Error	17	2,228.1			17	464.58		

**Figure 4:**
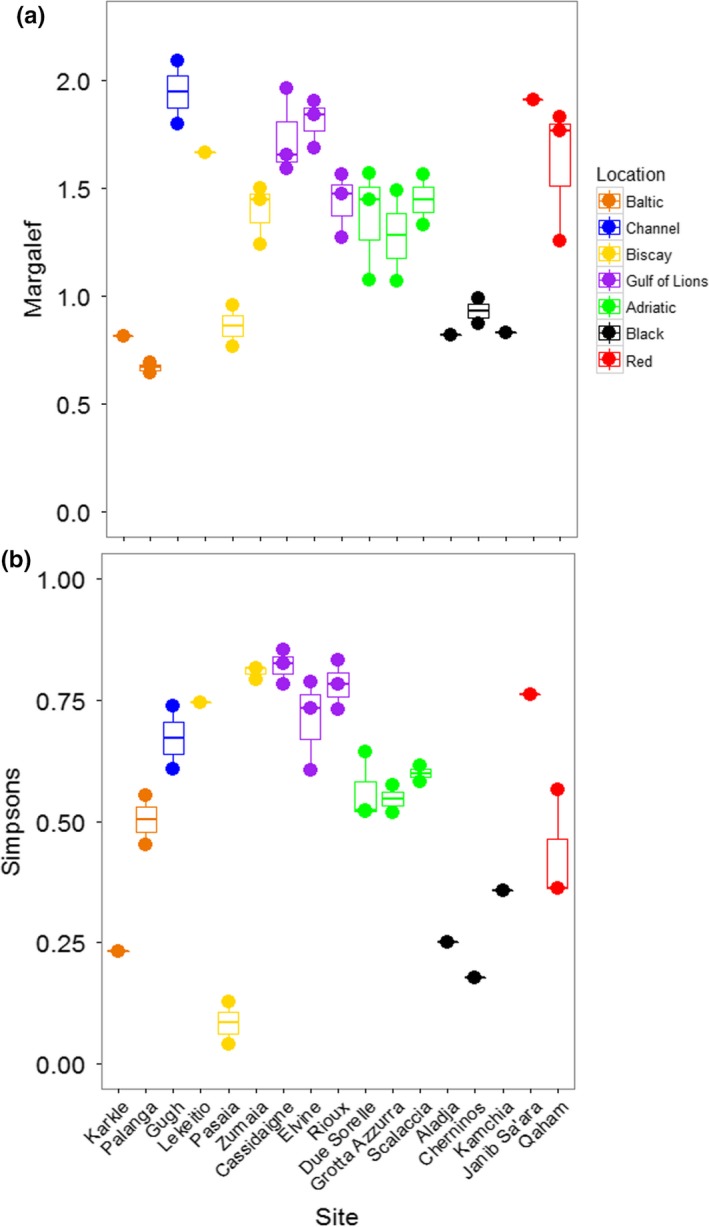
Taxonomic richness and diversity among sites based on morphological identifications. (a) Margalef's index of taxonomic richness. (b) Simpson's index of taxonomic diversity [Colour figure can be viewed at http://wileyonlinelibrary.com]

The PERMANOVA performed on Margalef's Index of taxonomic richness showed that richness varied significantly among locations, but did not vary among sites within locations (Table 3; Figure 4A). The Black and Baltic Seas showed lower levels of richness than other locations. Taxonomic diversity, based on Simpson's Index, showed the opposite pattern: locations were not different, but sites were different within locations (Table 3; Figure 4B). The Baltic and Red Seas, and especially the Bay of Biscay, showed a large variation in diversity among sites. Pasaia, a site found near a port in the Bay of Biscay, showed the lowest overall diversity due to an extreme abundance of amphipods at this site (Supporting Information Figure S1, Appendix S1).

**Table 3:** Richness and diversity. PERMANOVA of richness (left) and diversity (right) metrics among different locations. Top: morphological identifications to the lowest possible taxonomic level (usually class). Middle: all molecular operational taxonomic units (mOTUs) were considered. Bottom: mOTUs were collapsed to match the morphological data. Significant effects at *p* < 0.05 are highlighted in bold

Source of variation	Margalef's index of taxonomic richness				Simpson's index of taxonomic diversity			
	*df*	MS	pseudo‐*F*	*p*	*df*	MS	pseudo‐*F*	*p*
Morphological data								
Location	**6**	**0.689**	**7.027**	**0.005**	6	0.150	1.601	0.232
Sites (location)	10	0.089	2.265	0.063	**10**	**0.085**	**18.480**	**<0.001**
Error	18	0.039			18	0.005		
All mOTUs considered								
Location	**5**	**67.549**	**4.959**	**0.021**	**5**	**0.176**	**6.522**	**0.014**
Sites (location)	10	12.209	2.169	0.072	10	0.024	1.973	0.118
Error	17	5.628			17	0.012		
mOTUs collapsed to match morphological data								
Location	5	0.198	3.123	0.056	**5**	**0.187**	**6.565**	**0.017**
Sites (location)	10	0.058	1.01	0.477	10	0.026	1.278	0.323
Error	17	0.058			17	0.020		

### 3.2 Metabarcoding: mOTU identification

**Figure 5:**
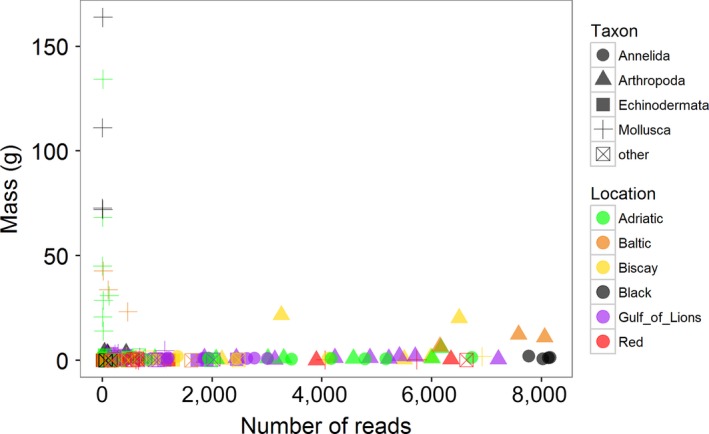
Correlation between the number of reads and the biomass of each phylum in the Artificial Substrate Unit (ASU). Data for both mass and read number was collapsed to the phylum level, such that each point represents a phylum in a given ASU (*N* = 5 groups; see Methods) within a sample. Colors represent seas; shapes represent phyla [Colour figure can be viewed at http://wileyonlinelibrary.com]

After running the bioinformatics pipeline, the analysis recovered 1,606 unique mOTUs from 403,958 quality filtered sequences. Of these, 242 (15.1%) were unable to be classified based on the reference database (BOLD). The remaining mOTUs were hierarchically classified using rdp and reported at the class/order level in this study where possible. For a given ASU, the correlation between the number of reads in a phylum and the biomass of that phylum was weakly negative (*r* = −0.132) and not significant (*p* = 0.09; Figure 5). This is due in large part to the very poor recovery of bivalve sequences. Bivalves contributed a great deal to the mass of samples, particularly in the Adriatic, Baltic, and Black Seas, as described above, but were largely absent from the mOTU list (Supporting Information Figure S1). When correlations between biomass and read number were performed for each phylum separately, only the annelids showed a significant correlation (*r* = 0.505, *p* = 0.003).

### 3.3 Metabarcoding: clustering and diversity analyses

The community composition of the ASUs based on raw mOTU data was significantly different among locations as well as among sites within locations (PERMANOVA, Table 2). An NMDS demonstrated strong separation among all locations (stress = 0.196; Figure 3B). When the molecular data were collapsed to match analyses conducted on the morphological dataset, community composition was different among locations, but not among sites within locations (PERMANOVA, Table 2). An NMDS analysis showed much greater overlap among locations at this lower taxonomic precision, with the Black, Baltic, and Red Seas separated from the other three locations, which largely overlapped (stress = 0.218, Figure 3C).

**Figure 6:**
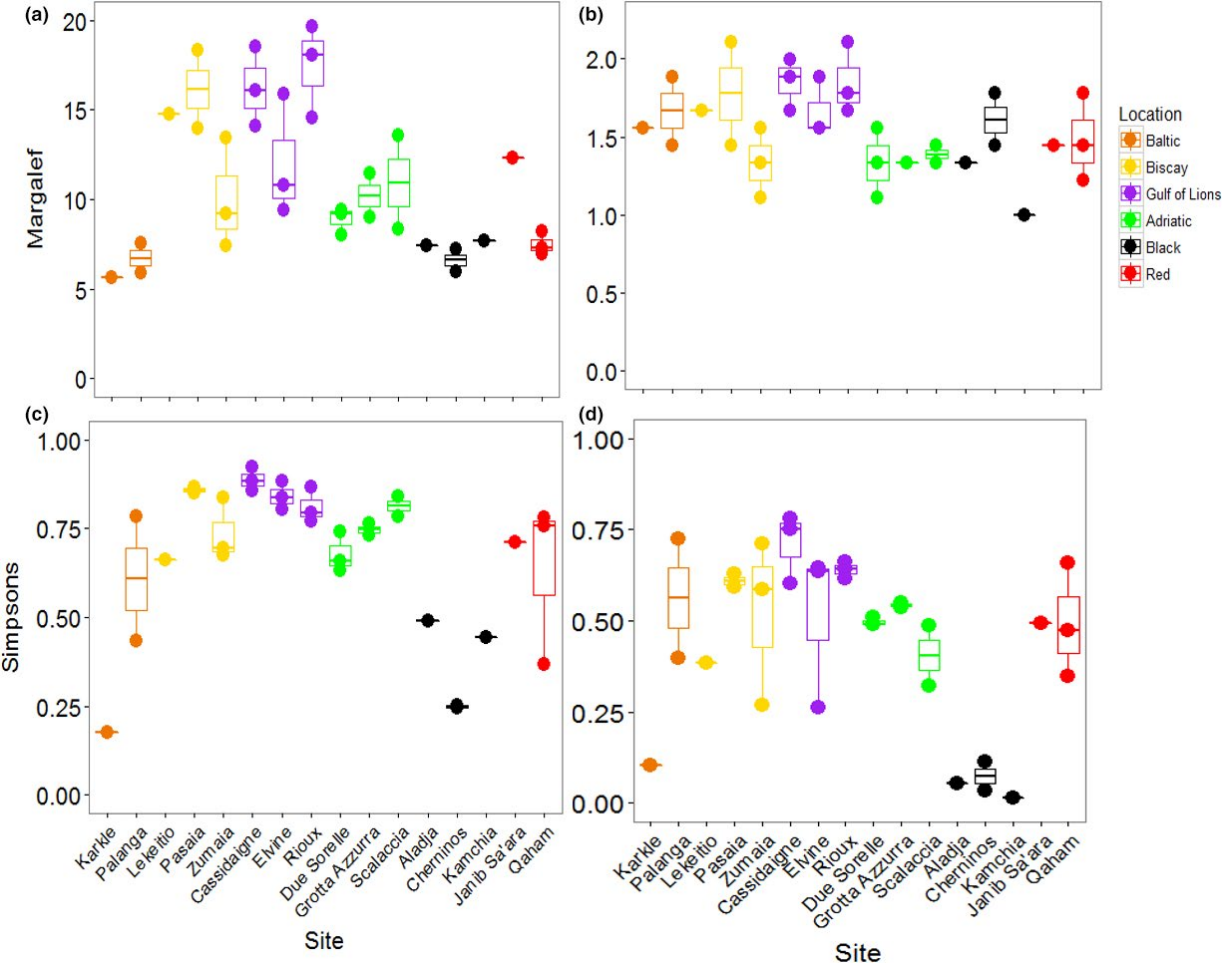
Taxonomic richness and diversity among sites based on molecular identifications. Margalef's index of taxonomic richness using (a) all molecular operational taxonomic units (mOTUs) and (b) mOTUs collapsed to match the morphological data. Note the difference in the *y*‐axis. Simpson's index of taxonomic diversity using (c) all mOTUs and (d) mOTUs collapsed to match the morphological data [Colour figure can be viewed at http://wileyonlinelibrary.com]

When considering all mOTUs, the taxonomic richness varied significantly among locations, but not among sites within locations (Table 3; Figure 6A). Richness was generally higher in the Bay of Biscay and the Gulf of Lions than other locations. Taxonomic diversity showed the same patterns (Table 3; Figure 6C). The Black Sea was noticeably less diverse than the other locations, although the site Karkle in the Baltic Sea also showed very low diversity. When mOTUs were collapsed to match morphological analyses, richness was not significantly different among locations or sites (Table 3; Figure 6B). Taxonomic diversity varied among locations, but not sites within locations (Table 3; Figure 6D). Sites in the Black Sea had lower diversity than other sites, and low diversity was again found at the site Karkle in the Baltic Sea (Figure 6D).

### 3.4 Comparison of methods

The differences among sites in community composition resulting from morphological and molecular approaches (Table 4) were significantly related, based on Mantel tests comparing dissimilarity matrices using Bray–Curtis distances. This was true both when all mOTUs were considered (Mantel's *r* = 0.638, *p* < 0.01) and when the molecular dataset was collapsed to match the morphological dataset (Mantel's *r* = 0.748, *p* < 0.01).

**Table 4:** Community dissimilarities among regions. Bray–Curtis measure of community dissimilarity based on morphological (above‐diagonal elements, italics) and molecular (below‐diagonal elements, all molecular operational taxonomic units considered) data. NA: not available. Numbers closer to 1 indicate higher dissimilarity between communities

	Baltic	Channel	Biscay	Gulf of Lions	Adriatic	Black	Red
Baltic		*0.530*	*0.610*	*0.723*	*0.626*	*0.457*	*0.572*
Channel	NA		*0.468*	*0.593*	*0.545*	*0.537*	*0.426*
Biscay	0.920	NA		*0.358*	*0.303*	*0.467*	*0.487*
Gulf of Lions	0.943	NA	0.852		*0.244*	*0.504*	*0.636*
Adriatic	0.918	NA	0.809	0.826		*0.325*	*0.581*
Black	0.882	NA	0.919	0.913	0.848		*0.535*
Red	0.891	NA	0.942	0.949	0.936	0.879	

**Figure 7:**
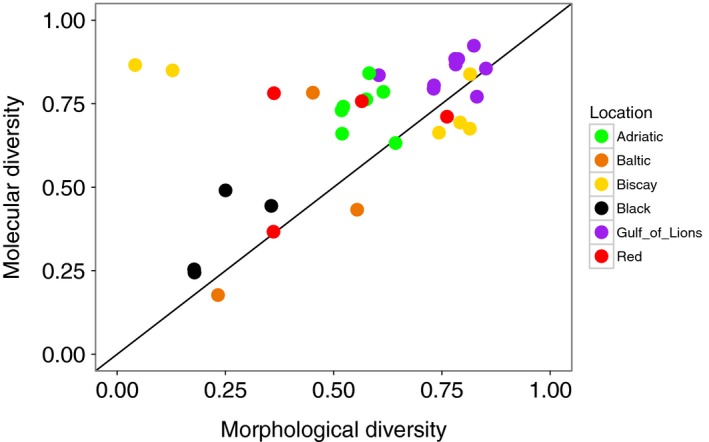
Correlation between morphological and molecular diversity. The correlation between taxonomic diversity measured with morphological data (Simpson's Index) and with molecular data (Simpson's Index, all molecular operational taxonomic units considered). The solid line represents a 1:1 relationship [Colour figure can be viewed at http://wileyonlinelibrary.com]

Diversity found with the molecular analysis (all mOTUs considered) was strongly correlated with the diversity obtained with the morphological analysis (*r* = 0.543, *p* = 0.001; Figure 7). The two replicates from Pasaia were outliers at the Biscay location in terms of high numbers of individuals and low diversity metrics based on morphological data (Figures 2, 4, 7). These samples were dominated by amphipods; pooling DNA in equimolar quantities removed this dominance and therefore diversity metrics calculated based on mOTUs at this site were similar to the rest of the Bay of Biscay (Figure 6). When these two outlying points were removed, the correlation increased (*r* = 0.783, *p* < 0.001). Diversity measured with mOTUs was generally higher than measured with morphological data, as indicated by a comparison to the 1:1 line (Figure 7).

## 4 DISCUSSION

### 4.1 Comparison of traditional and molecular approaches

The use of both morphological and metabarcoding approaches on the same set of samples allowed us to directly compare the two methods. Despite the overall similarity of the results found with the two datasets, there were some key differences. For instance, the clustering of mOTUs at 97% similarity resulted in a much higher number of taxonomic units than the morphological approach. The higher diversity observed with molecular data has previously been observed (e.g., Dell'Anno, Carugati, Corinaldesi, Riccioni, & Danovaro, 2015; Guardiola et al., 2016). While a higher diversity is observed in the molecular data, species assignments at the mOTU level are often currently unachievable. However, given the accuracy of the rdp classifier at coarse taxonomic scales, using a lower threshold for this parameter would allow the accurate assignment of taxonomy at the same high levels as those which were undertaken for the morphological data (i.e., class; Porter & Hajibabei, 2018). Finer classifications of the morphological data are achievable, but it would require a variety of taxonomic specialists with studies focused on smaller scales.

Diversity indices calculated using both approaches were highly correlated, and community composition patterns were similar between the morphological and molecular datasets based on Mantel tests performed on distance matrices. This correspondence in composition was observed both with the full molecular dataset and when the molecular data were collapsed to match the morphological data, indicating that this result is robust across various taxonomic levels in the molecular dataset.

Despite a correspondence in overall patterns between data types, metabarcoding did not recover all groups equally. For example, bivalves made up a large proportion of both the individuals and the biomass on the ASUs as measured with morphological data but were nearly absent in molecular results (Supporting Information Figure S1, Appendices S1 and S2). This may be due to low amplification success of bivalves using these universal primers. For instance, *Mytilus galloprovincialis*, a dominant species in the ASUs from the Adriatic but one that was unrecovered during molecular analysis, has a poor mismatch to the forward primers based on sequences available in GenBank. Lejzerowicz et al. (2015) found a similar undersequencing of molluscs relative to morphological data using metabarcoding techniques, albeit with a different gene (18S rRNA) and different primers. Metabarcoding of COI by Leray and Knowlton (2015) using the same primers as this study found few molluscs relative to annelids and arthropods, but their molecular results were not directly compared to morphological data. Kelly et al. (2017) found different groups of molluscs with morphological identification and COI metabarcoding based on eDNA samples and the Leray et al. (2013) primers. In contrast, Cowart et al. (2015) had a high sequencing rate of molluscs using Folmer primers and 454 sequencing. Ji et al. (2013) found that morphology and metabarcoding yielded similar conservation recommendations in geographically widespread locations; their genetic dataset was comprised of only arthropods, again measured using Folmer primers and 454 sequencing, highlighting the fact that not all metabarcoding protocols are alike. In particular, these two sets of primers (Folmer and Leray) were developed for different reasons and sequencing platforms, and may strongly impact the taxa recovered via metabarcoding.

Molecular data may also contain DNA from species in larger animals’ guts that was not sampled via morphological analysis. The inclusion of these gut contents can both allow us to sample taxa that are present in the community but not identifiable using morphology, and to sample animals that are not truly part of the ASU community. Distinguishing between these two cases, or even identifying a particular OTU as part of the gut contents of another organism, is not possible in this study.

Further anomalies were detected in the metabarcoding data. The two taxa that could clearly be identified as laboratory contamination (two species of *Abatus* urchins; see above) were removed prior to analyses. However, several potential anomalous taxa remained, particularly in the samples from the Baltic Sea. These samples yielded generally lower quantities of DNA compared to other locations and were the most difficult to amplify. The low quantity of DNA may have made these samples more prone to both sequencing errors and amplification of contaminants (the *Abatus* mOTUs were found in these samples, for example, although all samples were amplified at the same time). Most anomalous species in the Baltic samples, particularly those mOTUs that were identified as Mediterranean species and may represent cross‐contamination during the laboratory procedure, represented very low percentages of reads (<0.1%). It is unclear why the Baltic samples were the most difficult to amplify, as they were processed and stored in a manner identical to the other samples. It is possible that the DNA extractions in this region contained more PCR inhibitors. It is also likely that although the primers used were designed to amplify marine metazoans generally (Leray et al., 2013), the fauna of the Baltic may have more mismatches to the primers than fauna belonging to other seas, preventing reliable amplification.

Furthermore, the reference database used to identify mOTUs is limited: only species that have COI sequences in the database can be assigned to a taxon. As the reference databases are incomplete, sequenced mOTUs could actually be from organisms not present in the database. Many mOTUs could not be assigned beyond the phylum level, even in phyla where assignment to class level was possible for the morphological dataset. Filling the gaps in molecular databases will require collaboration between molecular ecologists and taxonomists (Bik, 2017). Given the difficulty of correctly diagnosing sources of error in mOTU identifications, we included all mOTUs except the two *Abatus* spp. in the analyses; this should not affect the overall validity of our clustering and diversity analyses. However, the uncertainty of correctly assigning taxonomy to mOTUs leads us to recommend caution in the use of metabarcoding to generate a precise species list. In addition, read number obtained with metabarcoding cannot be used as a substitute for measuring abundances or even biomass (Elbrecht & Leese, 2015; this study). These weaknesses are crucial to balance with the improved ability to detect species that may be difficult to identify in a morphological analysis (e.g., Pearman et al., 2016).

### 4.2 Biogeographical patterns

In addition to comparing methods, our large sampling zone allowed us to recover known biogeographic patterns from the marine environment. The seven locations investigated within these five regional seas showed different community composition (Supporting Information Figure S1, Figure 3). This was expected as the locations investigated ranged from the brackish, boreal Baltic Sea to the subtropical Red Sea. The seas considered vary in many factors, including geography, mean and seasonal temperatures, salinity, light availability, and nutrient levels. This separation was seen in the morphological data, although animals were only identified to the class level; it was also observed in the full metabarcoding dataset. When the molecular data were reanalyzed using the same level of taxonomic precision as the morphological data, the degree of separation among locations decreased (Figure 3C). However, at this level of taxonomic precision, there was still a clear separation between the Bay of Biscay, Adriatic Sea, and Gulf of Lions and the three peripheral locations (Baltic, Black, and Red Seas).

Both methods identified regional patterns of biodiversity that have been previously described in the literature, further confirming the efficacy of ASUs as a sampling device when combined with either morphological or molecular tools. For instance, we found a resemblance between the Basque coast (our sampling location in the Bay of Biscay) and the Mediterranean Sea. Fischer‐Piette (1935) first described the resemblance between these two regions: the Basque coast is more like the Mediterranean than other zones in the Bay of Biscay due to summer sea surface temperatures and other biogeographical and oceanographic conditions (Borja et al., 2004).

A second previously‐known pattern recovered in our data is the low diversity in the Baltic and Black Seas relative to other locations (Golemanski, 2007; Ojaveer et al., 2010; Zaitsev & Mamaev, 1997). The Black Sea consistently showed low diversity and richness, regardless of the dataset or metric considered. The Baltic Sea also showed lower taxonomic richness and diversity than other locations, but the diversity in the samples was more variable than the Black Sea. Due to unfavorable diving conditions which impeded recovery, the ASUs in the Baltic Sea were immersed for nearly twice as long as in the other locations. However, the similar patterns observed between the Baltic Sea and the Black Sea, where ASUs were recovered after 13 months, indicate that overall recruitment patterns are driven more by ecological and biogeographic conditions (comparatively small size of the regional species pool due to low salinity, geologically younger seas, smaller basin size) than deployment times (Ojaveer et al., 2010; Zaitsev & Mamaev, 1997).

## 5 CONCLUSION AND RECOMMENDATIONS

Metabarcoding using the COI gene shows great promise as a way to monitor marine biodiversity in hard‐substratum habitats, as diversity and composition metrics using metabarcoding and morphological data showed consistent results and patterns. However, based on the presence of several limitations and inconsistencies in the data, we conclude that the metabarcoding technique is not yet able to replace morphological identification as a monitoring tool in these habitats and make some future recommendations for researchers. First, we recommend the combined use of morphological and molecular approaches where possible; even our morphological analysis based at a low taxonomic resolution was able to identify limitations in our metabarcoding data. Second, we note that not all studies find the discrepancies that we have identified here, and urge researchers to collect preliminary data before implementing a metabarcoding‐based monitoring and conservation plan. Such preliminary data should take into account a project's overall goals: for instance, studies focusing on arthropods may have more success with the primer set used here than studies focusing on molluscs; studies in some locations may have greater overall success than in others (see our lower success in the Baltic Sea samples). Lastly, ASUs are small and inexpensive to deploy and process as compared to other monitoring techniques. Based on our success using them as sampling devices in hard‐bottom habitats, we recommend them for long‐term or high‐frequency monitoring.

## CONFLICT OF INTEREST

None declared.

## AUTHOR CONTRIBUTIONS

AEC conducted morphological identifications and molecular laboratory work. JKP conducted the bioinformatic analyses. PS and AEC conducted the statistical analyses. AEC and JKP drafted the manuscript. AB conceived the sampling design. All authors planned the study and contributed to the field sampling, sample processing, writing, and editing of the manuscript.

## DATA ACCESSIBILITY

Raw sequence reads have been deposited at the NCBI Sequence Read Archive (SRP093498). The datasets of taxon identification (morphological and molecular) are available as supplementary materials.
