# Supplementary material for: A comparative analysis of metabarcoding and morphology‐based identification of benthic communities across different regional seas
Source: Ecol Evol. 2018 Aug 13;8(17):8908–20. doi: 10.1002/ece3.4283 (PMC6157697; doi:10.1002/ece3.4283)
Supplement: Supplementary file 1 [file ECE3-8-8908-s001.docx]

**
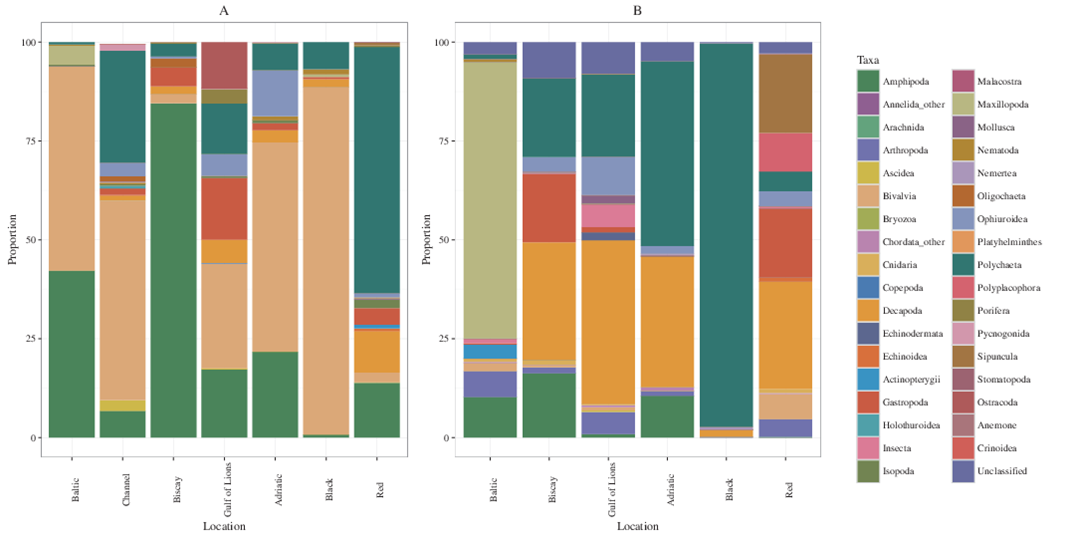
**

**Figure S1: Composition of ASUs.** Community composition plot based on morphological (A) and molecular metabarcoding (B) data in seven and six locations, respectively. Molecular data has been collapsed to match the morphological data, as described in the text. Each bar represents a location and the height of the stacked bars represent the relative proportion of each taxa, averaged across replicates within a location.
